# Intracapsular Versus Extracapsular Tonsillectomy: A Systematic Review and Meta-Analysis of Randomized Controlled Trials

**DOI:** 10.7759/cureus.115038

**Published:** 2026-08-23

**Authors:** Nada A Alshahrani, Razan M Althumali, Sarah Hadi S Atawi, Remas Ali Alshehri, Nada Alharbi, Abdulaziz Turki Alfadhel, Nada Mohammed Alghamdi, Sadeem Ibrahim Alqahtanii, Anfal Albaidani, Assaf A Alkathiri

**Affiliations:** 1 Otorhinolaryngology-Head and Neck Surgery, Asir Central Hospital, Abha, SAU; 2 Medicine and Surgery, Taif University, Taif, SAU; 3 Medicine and Surgery, University of Tabuk, Tabuk, SAU; 4 Medicine, University of Jeddah, Jeddah, SAU; 5 Medicine and Surgery, Al-Imam Mohammad Ibn Saud Islamic University, Riyadh, SAU; 6 Medicine, Taibah University, Madinah, SAU; 7 Audiology, King Khalid University, Abha, SAU; 8 Otolaryngology-Head and Neck Surgery, Riyadh Armed Forces Hospital, Ryiadh, SAU; 9 Otolaryngology-Head and Neck Surgery, Ministry of Health, Abha, SAU

**Keywords:** adult, extracapsular dissection, intracapsular dissection, meta-analysis, postoperative hemorrhage, postoperative pain, systematic review, tonsillectomy

## Abstract

Tonsillectomy is a common procedure associated with significant postoperative morbidity. Intracapsular tonsil surgery (ICTS), which preserves the tonsillar capsule, may reduce complications compared to traditional extracapsular tonsil surgery (ECTS). This systematic review and meta-analysis evaluates comparative outcomes of ICTS and ECTS, focusing on adult and mixed-age populations.

We conducted a systematic review following PRISMA guidelines, searching multiple databases for randomized controlled trials (RCTs) comparing ICTS and ECTS in adult and mixed-age populations (mean age ≥18 years). Primary outcomes were postoperative pain and hemorrhage; secondary outcomes included analgesia use and recovery. Data were pooled using meta-analysis where appropriate.

Four RCTs (n=231) were included. ICTS was associated with significantly lower postoperative pain scores and reduced analgesia requirements. The pooled risk of postoperative hemorrhage was significantly lower with ICTS (Risk Ratio 0.34, 95% CI 0.15-0.78, p=0.01). Recovery to normal diet and activity was faster following ICTS. The primary trade-off was a higher incidence of tonsillar remnants, though long-term symptom control remained effective.

ICTS may reduce postoperative pain, analgesic use, and hemorrhage risk compared with ECTS, though evidence is limited by small sample sizes, mixed-age populations, and heterogeneous techniques, warranting further adult-specific RCTs.

## Introduction and background

Tonsillectomy remains one of the most common surgical procedures performed globally, primarily to address recurrent tonsillitis, obstructive sleep apnea, and tonsillar hypertrophy [1]. Despite its frequency, it is associated with significant postoperative morbidity, including pain, hemorrhage, and delayed return to normal function, which profoundly impacts patient quality of life and healthcare utilization [1,2]. The traditional approach, extracapsular tonsil surgery (ECTS), involves complete removal of the tonsil and its capsule, exposing the underlying pharyngeal muscles [3]. While effective for disease eradication, this technique is linked to greater immediate postoperative pain and a higher risk of secondary hemorrhage due to the extensive surgical bed [3,4].

In contrast, intracapsular tonsil surgery (ICTS) involves the subtotal removal of the tonsillar parenchyma while preserving the underlying capsule [3,5]. This technique aims to reduce morbidity by protecting the pharyngeal musculature and neural structures, theoretically leading to less pain, lower analgesic requirements, and a decreased risk of bleeding [3,5,6]. However, concerns persist regarding the potential for tonsillar regrowth, residual symptoms, and the need for revision surgery, particularly in adult populations where disease patterns may differ from those in children.

The choice between ICTS and ECTS is a subject of ongoing clinical debate, driven by the need to balance efficacy against postoperative morbidity. Previous reviews and meta-analyses have often focused on pediatric populations or specific technologies, such as coblation [7-9]. A comprehensive synthesis focusing on adult patients, encompassing various intracapsular techniques (e.g., microdebrider, radiofrequency, powered resection) and their comparison to standard extracapsular methods, is necessary to guide evidence-based surgical decision-making.

Current guidelines lack specificity for adult patients, in whom pain control and bleeding risks are major concerns, and long-term outcomes data are more limited. Therefore, this systematic review and meta-analysis aimed to synthesize evidence from randomized controlled trials (RCTs) to evaluate the comparative effects of ICTS versus ECTS techniques in patients undergoing tonsillectomy, with a focus on adult and mixed-age populations. We specifically assessed critical outcomes, including postoperative pain, hemorrhage, recovery time, complication rates, and the need for revision surgery. This review seeks to inform clinical practice, enhance patient counselling, and identify key areas for future research in adult tonsil surgery.

## Review

Methods

This systematic review and meta-analysis were conducted and reported in accordance with the Preferred Reporting Items for Systematic Reviews and Meta-Analyses (PRISMA) guidelines [10]. The review was guided by this search question: among patients undergoing tonsillectomy for any indication, primarily adults but including mixed-age populations, including adult samples, what are the comparative effects of intracapsular versus extracapsular dissection techniques on postoperative pain, haemorrhage, recovery time, complications, and need for revision surgery, based on evidence from randomized controlled trials?

Eligibility Criteria

Studies were selected based on the following PICOS framework.

Population: adults, including mixed-age populations, including adult samples undergoing tonsillectomy for any indication (recurrent tonsillitis, obstructive symptoms, or hypertrophy) were included. Studies with mixed-age populations were included only if data for adults could be extracted separately or if the mean age was ≥18 years and the majority of participants were adults.

Intervention: the intervention included any ICTS, including but not limited to microdebrider-assisted, radiofrequency (tonsillotomy), powered intracapsular, or laser-assisted partial tonsillectomy.

Comparison: this included any ICTS, including cold steel dissection, electrocautery, or conventional total tonsillectomy.

Outcomes: primary outcomes were postoperative pain intensity and incidence of hemorrhage (primary and secondary). Secondary outcomes included analgesia requirements, time to return to normal diet and activities, complication rates (e.g., infection, nausea, otalgia), and need for revision surgery.

Study design: only published RCTs were considered for inclusion. Non-randomized studies, cohort studies, case series, and reviews were excluded.

Data Sources and Search Strategy

A comprehensive literature search was performed from inception until January 2025 across four electronic databases: PubMed/MEDLINE, Embase, the Cochrane Central Register of Controlled Trials (CENTRAL), and Web of Science. Only studies published in English were considered. The search strategy employed a combination of Medical Subject Headings (MeSH) terms and keywords related to the population (“adult,” “tonsillectomy”), intervention (“intracapsular,” "tonsillotomy,” “partial”), and study design (“randomized controlled trial”). Sample search terms included: (“tonsillectomy” OR “tonsil surgery”) AND (“intracapsular” OR “subtotal” OR “tonsillotomy” OR “partial”) AND (“randomized” OR “RCT”). The reference lists of all included studies and relevant review articles were manually screened to identify additional eligible trials. The complete database-specific search strategies are provided in the Supplementary file (Appendix A). 

Study Selection Process

**Figure 1:**
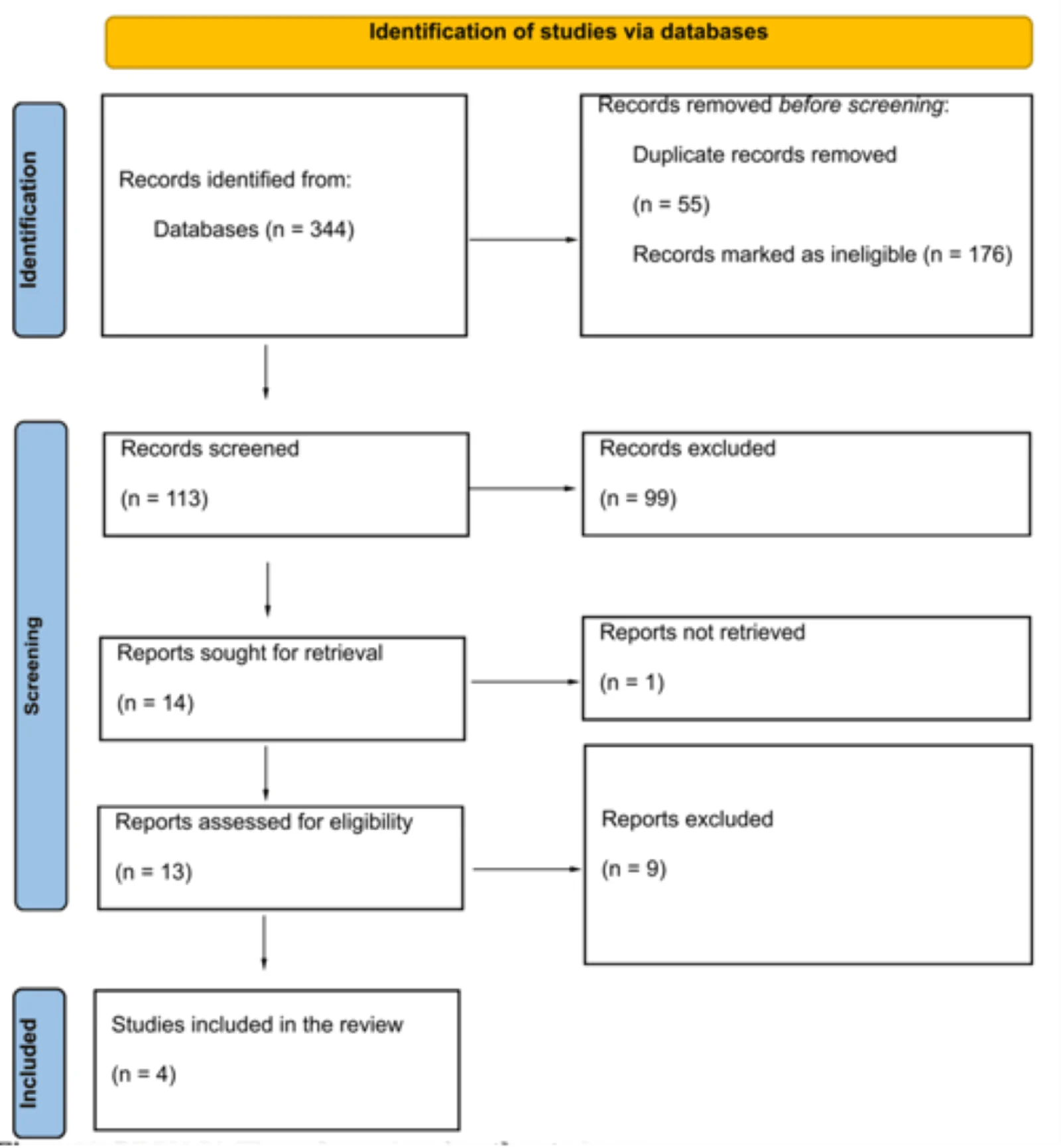
PRISMA Flow chart showing the study selection process.

Two independent reviewers screened all identified titles and abstracts against the eligibility criteria. Potentially relevant full-text articles were then retrieved and assessed independently by the same reviewers. Any discrepancies at either stage were resolved through discussion or, if necessary, by consultation with a third reviewer. The study selection process was documented using a PRISMA flow diagram (Figure 1).

Data Extraction and Management

Data were extracted independently by two reviewers using a standardized, piloted data extraction form. Extracted information included: study characteristics (first author, publication year, country, study design, sample size, age range/mean, and follow-up duration); patient characteristics (indication for surgery); intervention and comparator details (specific surgical techniques and technologies used); outcome data for continuous outcomes, such as pain scores (means, standard deviations, and sample sizes for each group at specified time points), and for dichotomous outcomes, such as hemorrhage, and complications (event counts and group totals).

Pain intensity was assessed using validated patient-reported outcome measures reported in the original trials. These included the Visual Analog Scale (VAS) [11], Numeric Pain Rating Scale (NPRS) [12], Faces Pain Rating Scale (FACES/FPRS) [13], and EuroQol Visual Analogue Scale (EQ-VAS) [14], SF-36 [15], and Tonsil and Adenoid Health Status Instrument (TAHSI) [16], depending on the study. Because different validated pain instruments were used across studies, standardized mean differences (SMDs) were calculated for quantitative synthesis when pooling was appropriate.

Risk of Bias Assessment

The methodological quality of each included RCT was assessed independently by two reviewers using the revised Cochrane Risk of Bias tool for randomized trials (RoB 2) [17]. This tool evaluates bias across five domains: 1) randomization process, 2) deviations from intended interventions, 3) missing outcome data, 4) measurement of the outcome, and 5) selection of the reported result. Judgments were categorized as low risk, some concerns, or high risk of bias. Disagreements were resolved by consensus.

Data Synthesis and Analysis

For dichotomous outcomes (e.g., hemorrhage), risk ratios (RR) with 95% confidence intervals (CIs) were calculated. For continuous outcomes (e.g., pain scores), standardized mean differences with 95% CIs were calculated to pool results from studies using different measurement scales. A fixed-effect model was used for meta-analysis when statistical heterogeneity was low (I² ≤ 50%); otherwise, a random-effects model was employed. Heterogeneity was quantified using the I² statistic, with values of 25%, 50%, and 75% representing low, moderate, and high heterogeneity, respectively.

Pre-specified subgroup analyses were planned to explore potential sources of heterogeneity based on: 1) surgical technology (e.g., microdebrider vs. radiofrequency), 2) primary indication for surgery (infectious vs. obstructive), and 3) length of follow-up (<1 year vs. ≥1 year). A meta-analysis was performed only for outcomes with data from at least 2 studies that were sufficiently homogeneous in terms of participants, interventions, and outcome measures. Where statistical pooling was not appropriate due to clinical or methodological heterogeneity, a narrative synthesis of findings was presented, structured by outcome domain. 

For the hemorrhage outcome, one of the study reported zero events in the intracapsular arm. A standard continuity correction of 0.5 was applied to the zero-event cell to allow inclusion in the pooled risk ratio estimate, per Mantel-Haenszel statistical method conventions.

Oubaid et al. [5] employed a within-patient (split-side) design, in which each participant received the intracapsular procedure on one side and the extracapsular procedure on the contralateral side, rendering outcomes non-independent between arms. Because standard risk ratio and mean-difference pooling methods assume independent groups, this study was excluded from all pooled meta-analyses (hemorrhage and pain), and its findings are reported narratively only, using the paired comparisons as analyzed in the original study.

Results

This review synthesized evidence from four RCTs comparing intracapsular and extracapsular tonsillectomy techniques in patients with recurrent tonsillitis, obstructive symptoms, or tonsillar hypertrophy. The outcomes of interest include postoperative pain, analgesia requirements, hemorrhage, recovery metrics, and complications.

Characteristics of Included Studies

Four RCTs were included, published between 2012 and 2025, conducted in Austria, Sweden, Iraq, and Japan. Sample sizes ranged from 11 to 104 patients. Follow-up periods varied from eight days to six years. Two studies focused exclusively on adults (Bender et al. [18]: 18-65 years; Wireklint et al. [19]: 16-25 years), while Oubaid et al. [5] and Sasano et al. [20] included mixed-age populations containing both pediatric and adult participants. All studies compared an intracapsular technique (microdebrider, radiofrequency, or powered resection) with an extracapsular technique (cold steel dissection, electrocautery, or conventional dissection) (Table 1).

**Table 1:** Characteristics of included studies. ICTS: intracapsular tonsil surgery, ECTS: extracapsular tonsil surgery, RCT: randomized controlled trial, TAHSI: tonsil and adenoid health status instrument, HRQL: health-related quality of life, SF-36: short form 36, EQ VAS: euroQol visual analogue scale, OSA: obstructive sleep apnea.

Study (Author, Year)	Country	Study Design	Total Participants (ICTS/ECTS)	Age Range (Mean ± SD)	Indication for Surgery	Intracapsular Technique (ICTS)	Extracapsular Technique (ECTS)	Follow-Up Duration	Primary Outcomes Assessed
Bender et al. 2015 [18]	Austria	RCT	104 (50/54)	18-65 years (26.4 ± 7.6)	Recurrent/chronic tonsillitis	Microdebrider-assisted ICTS	Cold steel dissection	Six months	TAHSI score, pain, hemorrhage, analgesia use
Wireklint et al. 2012 [19]	Sweden	RCT	76 (32/44)	16-25 years (25.7 ± 3.0)	Obstructive symptoms, recurrent infections	Radiofrequency tonsillotomy (TT)	Cold steel tonsillectomy (TE)	Six years	HRQL (SF-36, EQ VAS), hemorrhage, recovery, infection
Oubaid et al. 2018 [5]	Iraq	RCT (within-patient)	40 (same patients, both sides)	4-38 years (11.68 ± 7.44)	Tonsillar hypertrophy (grade 3-4)	Microdebrider intracapsular tonsillotomy (MT)	Cold steel dissection tonsillectomy (CDT)	14 days	Pain scores, otalgia, return to diet
Sasano et al. 2025 [20]	Japan	Single-blind RCT	11 (6/5)	5-60 years (PIT: 13.5 ± 11.0; cTE: 16.8 ± 18.1)	OSA or recurrent tonsillitis	Powered intracapsular tonsillectomy (PIT)	Conventional extracapsular tonsillectomy (cTE)	Eight days (pain/analgesia/diet); bleeding during follow-up	Pain scores, analgesia use, dietary intake, bleeding

Key Outcomes

Postoperative pain: all studies indicate that patients in the intracapsular group experienced significantly lower postoperative pain than patients in other groups. Bender et al. [18] found that pain medication usage was substantially lower in the ICTS group (p=0.049). Oubaid et al. [5] reported significantly reduced pain scores for microdebrider tonsillotomy from day 0 to day 10 (p<0.001 to p=0.009). Additionally, Sasano et al. [20] observed that powered intracapsular tonsillectomy resulted in lower pain scores on day 2 (p=0.036) with a similar trend noted over days 2 to 4 (p=0.053).

Analgesia requirements: reduced analgesic use was consistently observed in the intracapsular groups across the studies reviewed. Bender et al. [18] reported a significant difference in pain medication requirements (p=0.049). Furthermore, Sasano et al. [20] found that total analgesic doses were significantly lower in the PIT group (p<0.001).

Postoperative hemorrhage: post-tonsillectomy hemorrhage rates were generally low but significantly elevated in extracapsular tonsillectomy groups. Bender et al. [18] reported an overall hemorrhage rate of 21.2%, with ECTS at 29.6% and ICTS at 12% (p=0.03). Wireklint et al. [19] identified two primary and four secondary hemorrhages, all within the TE (tonsillectomy) group. In contrast, other studies by Oubaid et al. [5] and Sasano et al. [20] reported no hemorrhagic events.

Recovery: intracapsular tonsillectomy techniques are associated with faster patient recovery compared to traditional methods. According to Wireklint et al. [19], patients in the tonsillotomy group experienced fewer days of pain and a quicker return to diet and activities. Further, Oubaid et al. [5] confirm that intracapsular groups returned to a normal diet sooner. However, Sasano et al. [20] found no significant difference in dietary intake (p=0.389) between the groups. 

Complications: in terms of complications, tonsillar remnants were more common in the intracapsular groups, as indicated by Bender et al. [18], who reported 24 cases in the intracapsular group compared to four in the other group. Infection rates remained low and similar between both techniques, with additional complications such as nausea, vomiting, and otalgia occurring without consistent differences among the groups.

Table 2 illustrates key outcomes of intracapsular vs. extracapsular tonsillectomy. In general, intracapsular tonsillectomy techniques consistently reduce early postoperative pain, lower analgesic use, and decrease the risk of hemorrhage compared to extracapsular methods, leading to faster recovery. The primary disadvantage is a higher incidence of residual tonsillar tissue, though long-term symptom control remains effective.

**Table 2:** Key outcomes of intracapsular vs. extracapsular tonsillectomy. VAS: visual analog scale, ICTS: intracapsular tonsil surgery, ECTS: extracapsular tonsil surgery, TT: tonsillotomy, TE: tonsillectomy, MT: microdebrider tonsillotomy, CDT: cold dissection tonsillectomy,  PIT: powered intracapsular tonsillectomy, cTE: conventional tonsillectomy, NPRS: numeric pain rating scale.

Study (Author, Year)	Postoperative Pain (Scale and Findings)	Analgesia Requirements	Postoperative Hemorrhage	Recovery Metrics	Complications & Other Notes
Bender et al. 2015 [18]	Scale: 0-10, VAS finding: Pain medication use significantly lower in ICTS group (p=0.049)	Regimen: stepwise (paracetamol to naproxen to hydromorphone). Finding: significantly lower requirement in ICTS	Overall: 21.2% (22/104), ICTS: 12% (6/50), ECTS: 29.6% (16/54), Stat. Sig. p=0.03 (higher in ECTS)	General: rapid recovery suggested in ICTS group	Nausea/vomiting: 16 patients (6 ICTS, 10 ECTS). Tonsillar remnants: 28 patients (24 ICTS, 4 ECTS). Infection: None reported.
Wireklint et al. 2012 [19]	Scale: EQ VAS (self-rated health). Finding: no explicit pain scores; TT group had fewer days in pain	Not explicitly mentioned	TE group: 2 primary + 4 secondary hemorrhages (1 re-operated). TT group: 0 hemorrhages	TT group: fewer days in pain, quicker return to normal diet/activity.	Infection recurrence: TE 28%, TT 17% at 6 years (no significant difference). Other: 1 TT patient had post-op tonsillitis.
Oubaid et al. 2018 [5]	Scale: FACES (0-5) and NPRS (0-10). Finding: pain scores significantly lower for MT vs. CDT from Day 0-10 (p<0.001–0.009)	Regimen: oral paracetamol. Finding: prescribed as needed; use implied to be lower with MT	None reported in either group	MT group: pain-free sooner, quicker resumption of normal diet.	Otalgia: 70% of patients, correlated with CDT side. Infection: none reported.
Sasano et al. 2025 [20]	Scale: FACES pain rating scale (FRS). Finding: lower pain in PIT group on Day 2 (p=0.036); trend Days 2-4 (p=0.053)	Finding: total analgesic doses significantly reduced in PIT group (p<0.001)	None reported in either group	Dietary intake: no significant difference between groups (p=0.389).	General: No major complications reported. Note: very small sample size (n=11).

Meta-Analysis Results

Given the heterogeneity in outcome measures, follow-up durations, and patient ages, a meta-analysis was conducted only for dichotomous outcomes where data permitted.

**Figure 2:**
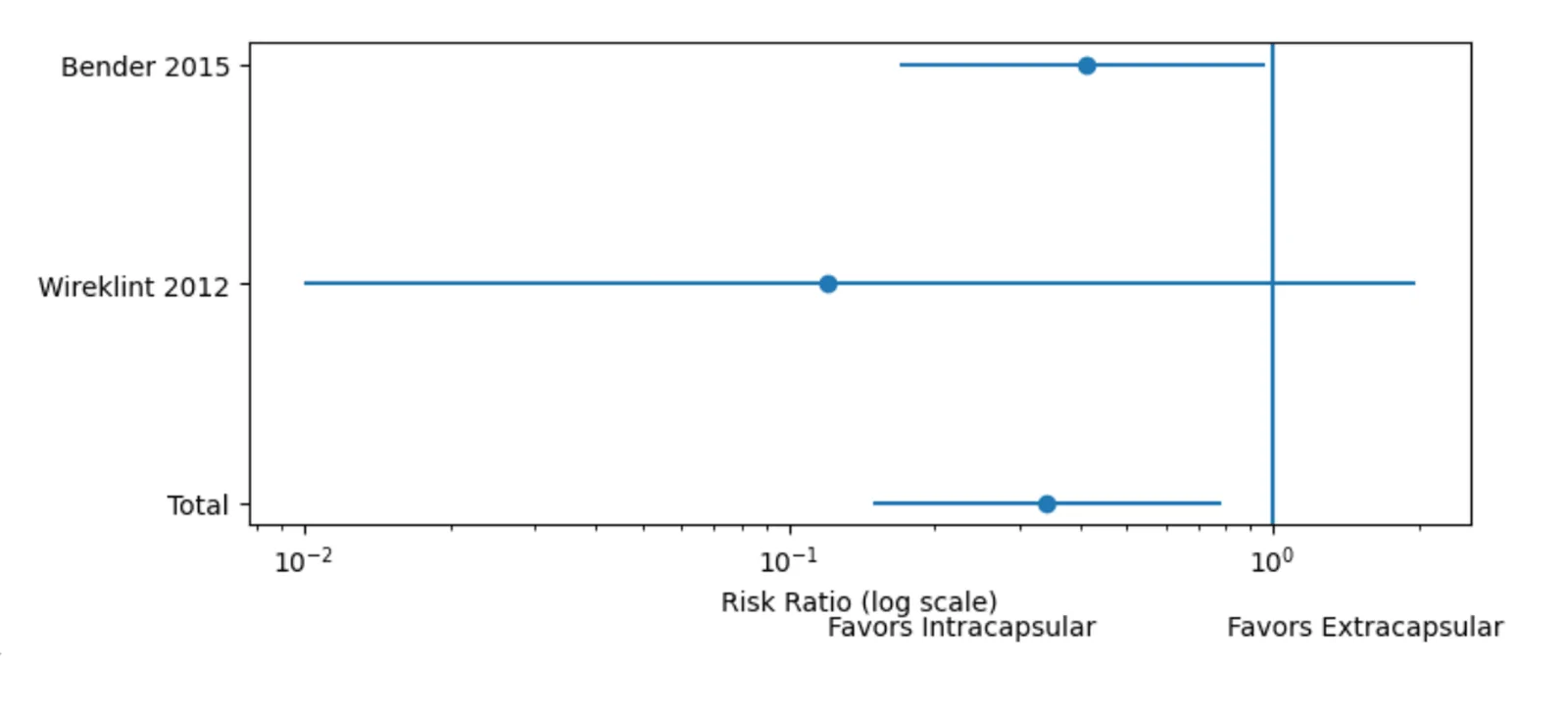
Forest plot comparing postoperative hemorrhage between intracapsular dissection tonsillectomy and extracapsular dissection tonsillectomy. Risk ratios are presented on a logarithmic scale with 95% confidence intervals. The analysis includes the studies by Bender et al. 2015 [18] and Wireklint et al. 2012 [19].

Postoperative hemorrhage: a fixed-effect model was used. Data from Bender et al. [18] and Wireklint et al. [19] were pooled. The risk ratio (RR) for hemorrhage in intracapsular vs. extracapsular groups was 0.34 (95% CI: 0.15-0.78), indicating a significantly lower risk of hemorrhage with intracapsular techniques, with no evidence of heterogeneity (I² = 0%) (Figure 2).

Postoperative pain (narrative synthesis): postoperative pain outcomes were not amenable to meta-analytic pooling due to heterogeneity in pain instruments (VAS, NPRS, FACES, EQ-VAS) and reporting formats across studies. Findings are therefore presented narratively. Only one intra-individual RCT by Oubaid et al. [5] provided extractable mean±SD values by postoperative day, so pooling was not possible. This study showed that on postoperative day 1, pain was lower on the intracapsular/microdebrider side than on the extracapsular/cold-dissection side (MD=-1.90; 95% CI:-2.29 to -1.51 on a 0-10 scale).

Subgroup Analysis

Pre-specified subgroup analyses by surgical technology (e.g., microdebrider vs. radiofrequency), indication for surgery (infectious vs. obstructive), and follow-up duration (<1 year vs. ≥1 year) were not performed. With only four included trials, each employing a distinct intracapsular technology, differing indications, and follow-up periods ranging from eight days to six years, there were insufficient studies within any stratum to permit meaningful statistical comparison.

Risk of Bias Assessment

**Figure 3:**
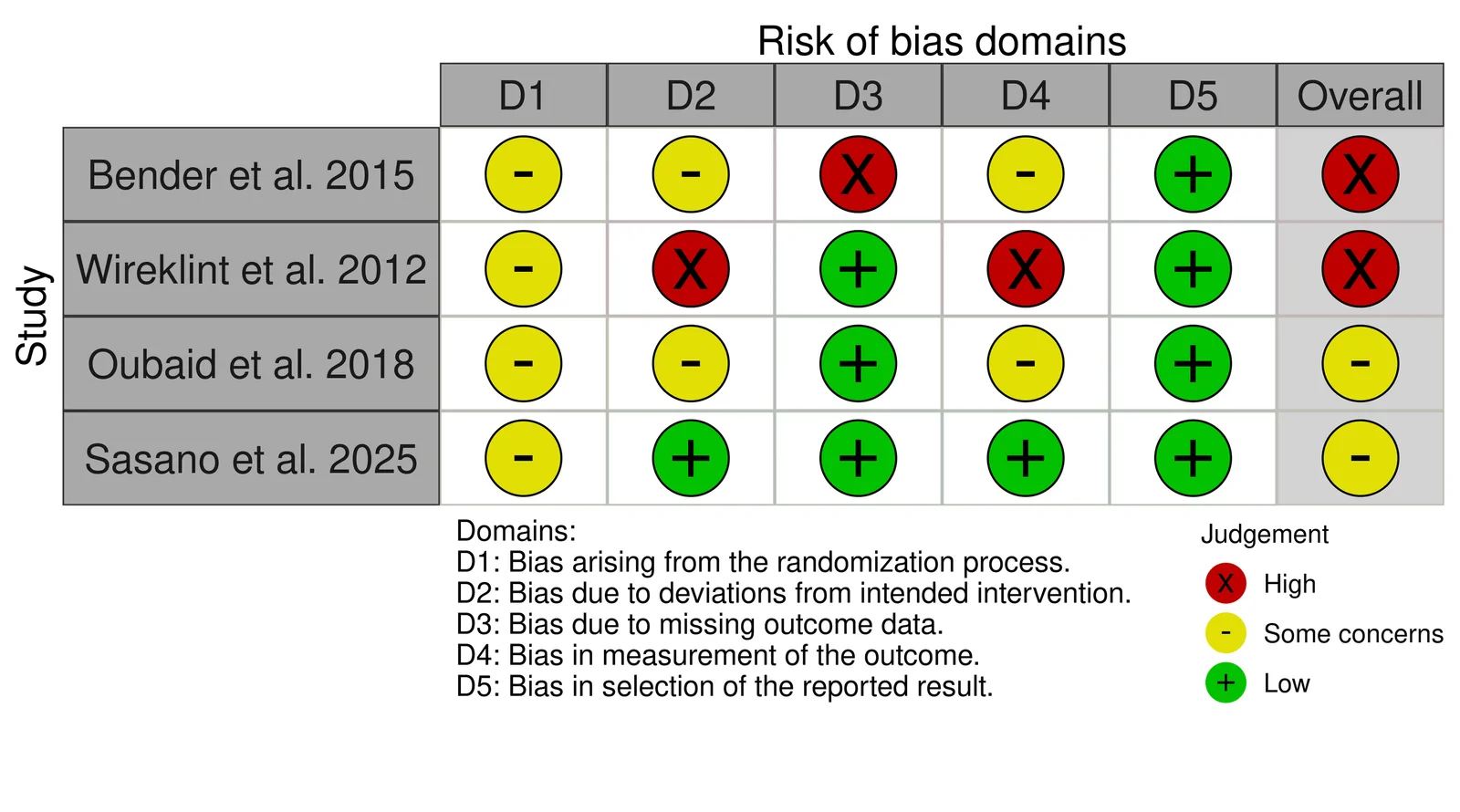
Risk of bias summary (RoB 2) [5,18-20].

Risk of bias was assessed using the Cochrane RoB 2 tool across five domains (Figure 3). All four studies showed some concerns in the randomization process domain, as allocation concealment was not explicitly described in any trial. Deviations from intended interventions varied by study: Sasano et al. [20] was rated low risk given explicit single-blinding of patients to procedure type, whereas Wireklint et al. was rated high risk given the absence of blinding combined with entirely self-reported subjective outcomes; Bender et al. and Oubaid et al. [5] were rated as having some concerns. Missing outcome data was low risk in three studies, but high risk in Bender et al. [18] due to 13% loss to follow-up at six months. Measurement of the outcome followed a similar pattern to the deviations domain, reflecting the same blinding limitations, with Wireklint et al. [19] rated high risk and Sasano et al. [20] rated low risk. Selective reporting was low risk across all four studies. Overall risk of bias was judged high for Bender et al. [18] and Wireklint et al. [19] and some concerns for Oubaid et al. [5] and Sasano et al. [20]. No study was judged overall low risk, reflecting the inherent difficulty of blinding in surgical trials and incomplete reporting of allocation concealment procedures across this literature.

Discussion

This systematic review and meta-analysis found that ICTS in adults is associated with significantly reduced postoperative pain, lower analgesic requirements, a decreased risk of secondary hemorrhage, and a faster return to normal activities compared with traditional ECTS. These outcomes are consistent with several previously published randomized controlled trials and large cohort studies, underscoring the clinical advantages of partial tonsillectomy techniques that preserve the tonsillar capsule.

Postoperative pain is one of the most prominent morbidities following tonsil surgery. In the present analysis, ICTS demonstrated lower pain scores and reduced analgesic usage compared with ECTS across several included trials. This observation mirrors findings from another systematic review that noted significantly less postoperative pain and shorter analgesic use following intracapsular tonsillectomy, using a variety of pain rating scales, although heterogeneity in measurement methods limited pooled analyses [9]. Similarly, a 2021 meta-analysis focusing specifically on intracapsular coblation tonsillectomy echoed these results, reporting significantly reduced late postoperative pain relative to extracapsular coblation techniques, suggesting that preservation of the tonsillar capsule may reduce the inflammatory response and nociceptive input from underlying pharyngeal musculature [7]. These data align with classical surgical principles that avoiding deeper dissection and exposure of sensory-rich muscle and nerve endings can mitigate postoperative discomfort, and they extend these principles to adult populations where pain and opioid use are important clinical considerations.

Another key finding of the current study is the reduced incidence of postoperative hemorrhage in ICTS compared with ECTS. Secondary bleeding events, which contribute to readmission and surgical reintervention, were consistently less common in intracapsular techniques. This is concordant with multiple large-scale observational analyses indicating lower hemorrhage rates with intracapsular approaches. For instance, Loh et al. [21] reported a significantly lower overall post-tonsillectomy bleed rate (1.7%) and markedly lower rates of return to theatre with intracapsular tonsillectomy compared with extracapsular techniques (4.1%) in a cohort of over 12,000 patients. Furthermore, recent meta-analytic data on intracapsular coblation tonsillectomy demonstrate overall hemorrhage rates around 1%, with very low proportions of primary and secondary hemorrhage events, supporting the safety profile of these techniques [7,8]. These findings are biologically plausible, as the retained tonsillar capsule may serve as a protective “biologic dressing” over the pharyngeal musculature and underlying blood vessels, decreasing exposure and vulnerability to delayed bleeding-a phenomenon well documented following extracapsular dissection where the surgical bed remains open for secondary intention healing.

Functional recovery following tonsil surgery, including resumption of diet and daily activities, was consistently faster in ICTS groups. This concurs with results from a randomized clinical trial comparing laser tonsillotomy (a form of intracapsular surgery) to traditional tonsillectomy in adults, in which patients in the tonsillotomy arm returned to full functional recovery significantly earlier and reported shorter pain duration and shorter analgesic use [22]. Faster recovery has also been documented in pediatric and mixed-population reviews, with improved oral intake and activity levels seen within days after intracapsular tonsil surgery compared with total removal [9]. Such consistent benefits across study designs and age groups reinforce the generalizability of these findings.

One recognized concern with intracapsular procedures is the presence of tonsillar remnant tissue and its potential regrowth. In adult cohorts, recent longitudinal follow-up studies report modest regrowth rates (about 3.9%) and very low reoperation rates, indicating that, while residual tissue persists, clinical significance may be limited for many patients [23]. This aligns with pediatric literature showing low regrowth requiring revision, though the clinical implications may differ based on the indication for surgery (e.g., recurrent tonsillitis versus obstructive sleep-disordered breathing) and patient age. For example, some long-term tonsillotomy data indicate that symptom recurrence is slightly higher than in total tonsillectomy, though these differences are small and not always statistically significant [24].

Patient selection is a critical factor when considering tonsil surgery techniques. While intracapsular methods appear advantageous for postoperative morbidity, especially pain and bleeding, traditional extracapsular tonsillectomy remains the historical standard for conditions characterized by severe or frequent recurrent tonsillitis, where complete lymphoid removal might theoretically reduce disease recurrence. However, the present study’s findings, aligned with broader literature, suggest that the difference in infection recurrence between techniques is minimal when evaluated within available follow-up periods [3]. This supports shared decision-making tailored to individual patient priorities, balancing considerations of recovery speed, complication risk, and long-term disease control.

The findings of this review should be interpreted in the context of existing study limitations. As in prior systematic assessments, heterogeneity in surgical methods (e.g., microdebrider, coblation, radiofrequency, laser), pain assessment tools, and follow-up durations complicates direct comparisons across studies and may affect the strength of pooled estimates [9]. Additionally, many existing trials lack long-term follow-up data, particularly for adult populations, limiting our understanding of durable symptom control and regrowth outcomes. Blinding challenges inherent in surgical trials further introduce the possibility of performance and detection bias. Two of the four included trials enrolled mixed pediatric-adult populations without separately reported adult outcomes, which limits the precision of conclusions specific to adult patients. These trials were retained given the scarcity of adult-only RCTs in this literature, but their inclusion means pooled and narrative findings should be interpreted as applicable to a predominantly young, mixed-age population rather than an exclusively adult one.

Future research should prioritize adequately powered, multicenter randomized controlled trials in primarily adult cohorts to validate these findings with standardized outcome measures, including long-term outcomes such as tonsillar symptom recurrence and quality-of-life metrics. Economic evaluations that incorporate both direct and indirect costs could further clarify the cost-effectiveness of intracapsular approaches, particularly in healthcare systems where postoperative morbidity has substantial economic implications. Moreover, subgroup analyses based on the indication for surgery (e.g., recurrent tonsillitis vs obstructive symptoms) may help refine clinical guidelines on the optimal surgical technique.

## Conclusions

ICTS may reduce postoperative pain, analgesic use, and hemorrhage risk compared with ECTS. However, the evidence is limited by small sample sizes, mixed-age populations in two of the four included trials, and heterogeneous surgical techniques. Concerns regarding tonsillar remnant persistence and regrowth remain, though available follow-up data suggest limited long-term clinical impact. Further adult-specific randomized controlled trials, with standardized outcome measures and longer follow-up, are needed to confirm these findings and guide surgical decision-making.

## Appendices

Appendix A

Supplementary file of full electronic search strategies.

**Table 3:** Supplementary table of full electronic records.

Database	Search strategy
1. PubMed/MEDLINE	( "Tonsillectomy"[Mesh] OR tonsillectom\*[tiab] OR "tonsil surgery"[tiab] OR "tonsil surgeries"[tiab] OR tonsillotom\*[tiab] ) AND ( intracapsular[tiab] OR "intra-capsular"[tiab] OR subtotal[tiab] OR partial[tiab] OR tonsillotom\*[tiab] OR "partial tonsillectomy"[tiab] OR "subtotal tonsillectomy"[tiab] ) AND ( "Adult"[Mesh] OR adult\*[tiab] ) AND ( "Randomized Controlled Trial"[Publication Type] OR randomized[tiab] OR randomised[tiab] OR randomly[tiab] OR trial[tiab] OR "controlled trial"[tiab] )
2. Embase (Elsevier)	( 'tonsillectomy'/exp OR tonsillectom\*:ti,ab,kw OR 'tonsil surgery'\:ti,ab,kw OR 'tonsil surgeries'\:ti,ab,kw OR tonsillotom\*:ti,ab,kw ) AND ( intracapsular\:ti,ab,kw OR 'intra-capsular'\:ti,ab,kw OR subtotal\:ti,ab,kw OR partial\:ti,ab,kw OR tonsillotom\*:ti,ab,kw OR 'partial tonsillectomy'\:ti,ab,kw OR 'subtotal tonsillectomy'\:ti,ab,kw ) AND ( 'adult'/exp OR adult\*:ti,ab,kw ) AND ( 'randomized controlled trial'/de OR random\*:ti,ab,kw OR trial\:ti,ab,kw OR 'controlled trial'\:ti,ab,kw ) AND [english]/lim AND [embase]/lim
3. Cochrane CENTRAL	#1 MeSH descriptor: [Tonsillectomy] explode all trees #2 (tonsillectom\* OR "tonsil surgery" OR "tonsil surgeries" OR tonsillotom\*)\:ti,ab,kw #3 #1 OR #2 #4 (intracapsular OR "intra-capsular" OR subtotal OR partial OR tonsillotom\* OR "partial tonsillectomy" OR "subtotal tonsillectomy")\:ti,ab,kw #5 #3 AND #4 #6 MeSH descriptor: [Adult] explode all trees #7 (adult\*)\:ti,ab,kw #8 #6 OR #7 #9 (random\* OR trial OR "controlled trial")\:ti,ab,kw #10 #5 AND #8 AND #9
4. Web of Science Core Collection	TS=( tonsillectom\* OR "tonsil surgery" OR "tonsil surgeries" OR tonsillotom\* ) AND TS=( intracapsular OR "intra-capsular" OR subtotal OR partial OR tonsillotom\* OR "partial tonsillectomy" OR "subtotal tonsillectomy" ) AND TS=(adult\*) AND TS=( random\* OR "randomized controlled trial" OR "randomised controlled trial" OR trial OR "controlled trial" )
